# Genome and transcriptome-wide study of carbamoyltransferase genes in major fleshy fruits: A multi-omics study of evolution and functional significance

**DOI:** 10.3389/fpls.2022.994159

**Published:** 2022-11-03

**Authors:** Yogeshwar V. Dhar, Mehar H. Asif

**Affiliations:** ^1^ CSIR-National Botanical Research Institute (CSIR-NBRI), Lucknow, India; ^2^ Academy of Scientific and Innovative Research to Academy of Scientific and Innovative Research (AcSIR), Ghaziabad, India

**Keywords:** carbamoyltransferase, fleshy fruit, fruit ripening, methylation, evolution

## Abstract

The carbamoyltransferase or aspartate carbamoyltransferase (ATCase)/ornithine carbamoyltransferase (OTCase) is an evolutionary conserved protein family, which contains two genes, ATCase and OTCase. The ATCase catalyzes the committed step in the synthesis of UMP from which all pyrimidine molecules are synthesized. The second member, OTCase, catalytically regulates the conversion of ornithine to citrulline. This study traces the evolution of the carbomoyltransferase genes in the plant kingdom and their role during fruit ripening in fleshy fruits. These genes are highly conserved throughout the plant kingdom and, except for melon and watermelon, do not show gene expansion in major fleshy fruits. In this study, 393 carbamoyltransferase genes were identified in the plant kingdom, including 30 fleshy fruit representatives. Their detailed phylogeny, evolutionary patterns with their expression during the process of fruit ripening, was analyzed. The ATcase and OTcase genes were conserved throughout the plant kingdom and exhibited lineage-specific signatures. The expression analysis of the ATcase and OTcase genes during fruit development and ripening in climacteric and non-climacteric fruits showed their involvement in fruit ripening irrespective of the type of fruits. No direct role in relation to ethylene-dependent or -independent ripening was identified; however, the co-expression network suggests their involvement in the various ripening processes.

## Introduction

The carbamoyltransferase or ATCase/OTCase enzymes, member of transcarbamylase protein family (Couchet et al., 2021), are universal in their occurrence, from prokaryotes to eukaryotes (Couchet et al., 2021). They play an important role in an organism’s biology by regulating the urea cycle (Smith and Garg, 2017), *de novo* pyrimidine biosynthesis (Schröder et al., 2005), and arginine biosynthesis (Winter et al., 2015; Urbano-Gamez et al., 2020), thus influencing the growth- and development-related processes. Their functional involvement in several important biological processes makes them crucial molecules for agronomic improvement of crop plants. The carbamoyltransferase contains two genes: aspartate carbamoyltransferase (ATCase) and ornithine carbamoyltransferase (OTCase). These two genes share different nucleotide and protein sequences but substantially conserved protein fold/structure. The ATCase genes participate in the enzymatic conversion of aspartate and carbamoyl phosphate into the N-carbamoyl-L-aspartate (CAA). This conversion is the initiation step of the pyrimidine biosynthetic pathway. The second member, OTCase, plays a crucial role in urea cycle by catalyzing the ornithine and carbamoyl phosphate to citrulline. OTCase is also known for its role in arginine biosynthesis and other nitrogenous compound in cytosol (Urbano-Gamez et al., 2020).

The ATCase consists of a catalytic homotrimer with three active sites in between the subunits, which can be allosterically regulated by association with other proteins. Many studies have been done on the biochemical and structural properties of prokaryotic, fungal, and animal ATCases (Wild et al., 1988; Kollman and Doolittle, 2000; Hong et al., 2004; Serre et al., 2004; Rabinowitz et al., 2008). There are limited studies in plants, and recently, the structural and functional analysis of *Arabidopsis* ATCase has been done (Bellin et al., 2021). They have shown a UMP-based regulation of the ATCases exclusively found in plants.

Apart from the functional role, the evolutionary aspect for the functional divergence and specificity in plant carbamoyltransferase genes is not well documented. Here, we identified members of carbamoyltransferase genes from various plant genomes with evolutionary divergence in their signature and focused on the representatives of fleshy fruits, with their transcriptomic and methylomic expression, to understand their behavior and influence during the process of fruit ripening. The stage-specific regulation of ethylene mediated fruit ripening, and its linkage with polyamine pathway is an important aspect of the fruit development process, where the carbamoyltransferase plays a crucial role with their ability to regulate polyamines. The involvement and essential role of polyamines in fruit ripening are well evidenced at physiological and molecular levels, and the regulation of polyamines shows the system’s gene regulation for essential steps of fruit ripening in climacteric and non-climacteric fruits through the carbamoyltransferase machinery (Fortes and Agudelo-Romero, 2018; Gao et al., 2021).

## Material and methods

### Identification of carbamoyltransferase members in plant genomes

The carbamoyltransferase genes were identified by using the *Arabidopsis* and rice sequences as input seeds for major plant genomes used in study of Zhao et al. (Zhao and Schranz, 2019), applying a two-step identification process. In step 1, the BLAST program (Altschul et al., 1990) was used to find the carbamoyltransferase genes in plant genome sequence databases, including the genome-dedicated databases, along with phytozome (https://phytozome-next.jgi.doe.gov/) and plant ensemble (https://plants.ensembl.org/). In step 2, the HMMER profile was constructed, and sequences were searched on the basis of profile HMM (Eddy, 2011). All the retrieved sequences were checked by the SMART database (http://smart.embl-heidelberg.de/) (Letunic et al., 2015) and CDD (Marchler-Bauer et al., 2007) for the presence of conserved ATcase/OTcase domains. Sequences lacking the conserved domain were excluded from further analysis.

### Phylogenetic analysis, microsynteny, and conserved motif search

Identified sequences of the ATcase/OTcase proteins from different plant groups were aligned using Clustal X (Thompson et al., 2002), with a gap opening penalty of 10 and a gap extension penalty of 0.1. Phylogenetic relations of ATcase/OTcase genes were analyzed using the IQ-TREE tool (Minh et al., 2020) maximum likelihood method with the JTT model for amino acids with ultrafast bootstrapping value of 1,000 and were considered as the parameters. To identify the signature splitting between two members of ATcase/OTcase, the split network analysis has been performed and visualized using the SplitsTree (Huson and Bryant, 2006) program. This complete process was used for kingdom-wide identified sequences and sequences identified in fruit representatives. The microsynteny analysis has been performed to identify the syntenic clusters between the different groups using the SynNet pipeline (Zhao et al., 2017). To understand the substitution, pressure with their time of divergence was estimated by calculating the synonymous substitution and non-synonymous substitution rate using the kS/kA calculator utility of tbtools (Chen et al., 2020). The speciation time in kS/kA analysis was obtained from timetree.org. To find out the conserved *de novo* sequential motif in ATcase/Otcase sequences, an offline MEME program (Bailey et al., 2009) was employed.

### Transcriptome and methylome expression analysis

The transcriptomic meta-analysis was performed for nine different fruits. The expression of ATcase/Otcase genes from six fruits (apple, banana, grape, papaya, melon, and strawberry) were extracted from the GEO file of fruit encode project GSE116581 (Lu et al., 2018). For watermelon and pineapple, the expression values were extracted from their expression databases (http://cucurbitgenomics.org/rnaseq/ home for watermelon and http://pineapple.angiosperms.org/pineapple/html/index.html for pineapple ). The *Citrus sinensis* fruit expression was extracted from GSE125726 (Feng et al., 2019). The differential expression (DE) of fpkm values were calculated using the DEseq (Anders and Huber, 2010) package and plotted using the MeV tool (Howe et al., 2011). The details of transcriptomic data used for analysis are mentioned in tabular form below (**Table 1**).

**Table 1:** The fruit stage and sample wise detail of transcriptomic data, used for expression analysis.

Plant	GEO/data accession	Sample	Stage
Apple	GSE116581 (Fruit Encode Project)	Pulp	Unripe and ripe
Banana	GSE116581 (Fruit Encode Project)	Peel and pulp	Unripe green and ripe yellow
Grape	GSE116581 (Fruit Encode Project)	Fruit flesh	Immature green and ripe; Green and breaker
Papaya	GSE116581 (Fruit Encode Project)	Pulp	30 DPA and 150 DPA
Melon	GSE116581 (Fruit Encode Project)	Fruit flesh	10 DPA and 30 DPA
Strawberry	GSE116581 (Fruit Encode Project)	Receptacles	Unripe and ripe
Orange	GSE125726	Juice sac	Stage 1 green, stage 3 break, stage 6 ripe
Watermelon	SRP012849	Fruit flesh	10 DPA and 34 DPA
Pineapple	Pineapple genome database	Fruit	Stage 1 green, stage 3 golden breaker, stage 5 ripe golden

To understand the epigenetic influence of methylation and related regulation in ATcase genes, the bisulfite sequencing data, available in the fruit encode project (GSE116581), were considered for four representative fruits, namely, apple, banana, grape, and strawberry. For methylation analysis, the epigenetic analysis module of CLC genomics workbench (https://digitalinsights.qiagen.com/) was used with default values. The genomic coordinates were mapped on respective reference genome. The total methylation level with CG, CHG, and CHH contexts were calculated with a window length of 100 and *p*-value of 0.05, along with differently methylated regions using the epigenetic analysis module in CLC genomics workbench.

### Gene co-expression network analysis

The relative co-expression profiles of ATcase/OTcase genes, for four fruits, were calculated using Pearson’s correlation method implemented in the expression correlation module of the Cytoscape tool for their correlation value (R-value) using the fpkm values. The identified ATcase genes were used as bait genes for identification of co-expressing genes. The co-expression network was visualized and analyzed in the Cytoscape tool (Shannon et al., 2003).

### Protein structural analysis

To understand and compare the structural features of ATcase protein, the protein structural modeling was performed using the Phyre2 tool (Kelley et al., 2015). The five plant representative sequences from algae (*Gonium pectorale*), bryophyte (*Marchantia polymorpha*), pteridophyte (*Selaginella moellendorffii*), gymnosperm (*Thuja plicata*), and angiosperm (*Arabidopsis thaliana*) were considered for structural modeling. The bacterial protein model was downloaded from the RCSB-PDB database (www.rcsb.org/) with ID number 5vmq.

## Results and discussion

### Identification of aspartate carbamoyltransferase/ornithine carbamoyltransferase proteins in plant groups

A total of 520 ATcase/OTcase genes (plant kingdom) were retrieved from the different plant groups, mentioned in Zhao et al. (Zhao and Schranz, 2019). The sequences were identified using blast and profile HMM method, using *Arabidopsis* and rice ATcase/OTcase sequences as seed. After removal of partial and redundant sequences, 393 sequences in total were considered as final sequences (**Supplementary Table S1**). From these sequences, 98 full-length sequences (**Supplementary Table S2**) with complete domain coverage in major plant species were considered as representative sequences and were used for further analysis. The occurrence of these genes in plant groups clearly indicated toward their conserved nature, as we observe two members of ATcase/OTcase in maximum plant groups and very few plant representatives showed presence of more carbamoyltransferase genes. In major fruit representatives (**Table 2**), the highest number was observed in melon (11 members) and watermelon (4 members), whereas apple, banana, grape, etc., showed two to three members only. The increased members could be due to either the whole genome duplication events or the expansion because of duplication. In a study in prokaryotic carbamoyltransferase, there was evidence of duplication of the genes and loss after duplication, thus maintaining the gene number. This could have also happened in plants where multiple rounds of whole genome duplications have taken place but the number of the genes is limited to two or three only.

**Table 2:** The fruits selected for study and number of carbamoyltransferase genes identified in them.

Fruit	Gene
Apple	3
Banana	2
Melon	11
Strawberry	2
Papaye	2
Citrus	2
Pineapple	2
Watermelon	4
Grape	2

### Phylogenetic evolutionary analysis and structural comparison of aspartate carbamoyltransferase/ornithine carbamoyltransferase members

**Figure 1:**
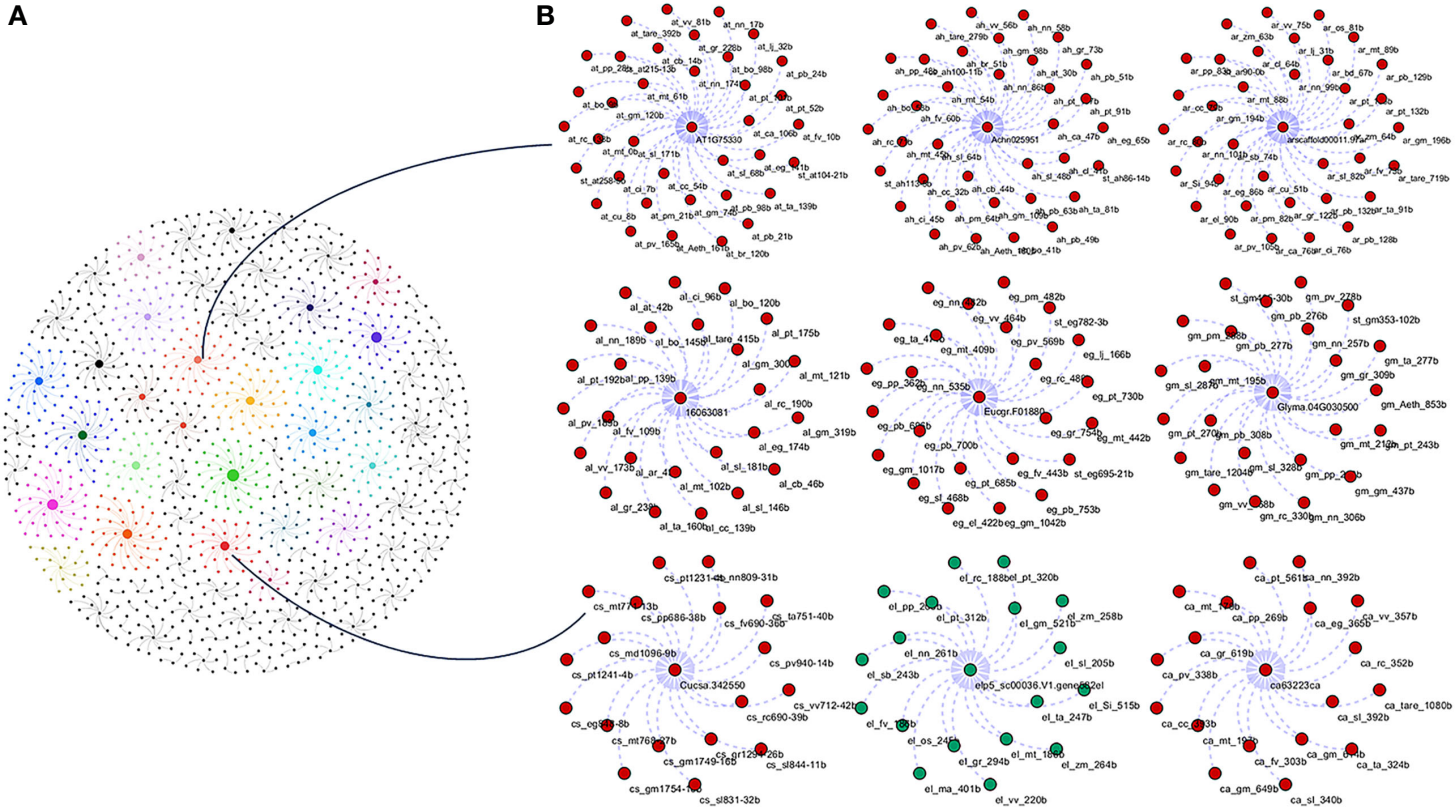
Kingdom-wide microsyntenic network of carbamoyltransferase genes, showing the major syntenic hubs in plant groups for aspartic and ornithine carbamoyltransferase genes. **(A)** The kingdom-wide microsyntenic network in globe net layout, where different colors represent different lineage-specific groups and node size represents connected syntenic genes. **(B)** The zoomed representation of lineage-specific groups showing major hubs, where the red color is for dicots and the green color is for monocot nodes.

**Figure 2:**
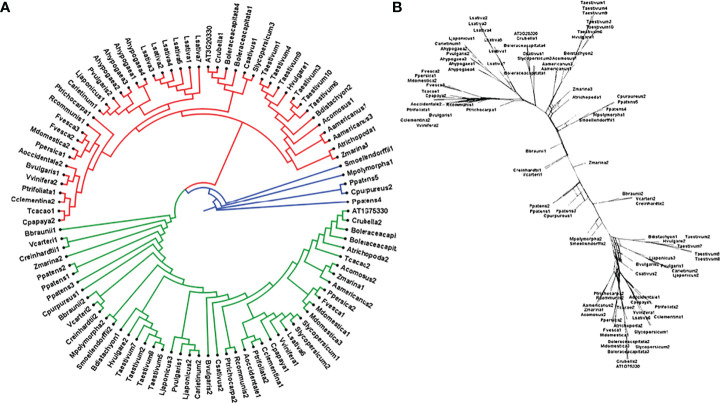
Phylogenetic **(A)** tree of carbamoyltransferase genes in plant groups including fruit representative members, where the red clade is aspartate carbamoyltransferase and the green clade is ornithine carbamoyltransferase. Basal groups are represented by the blue color. **(B)** The sequence signature splitting is represented by the phylogenetic split network.

**Figure 3:**
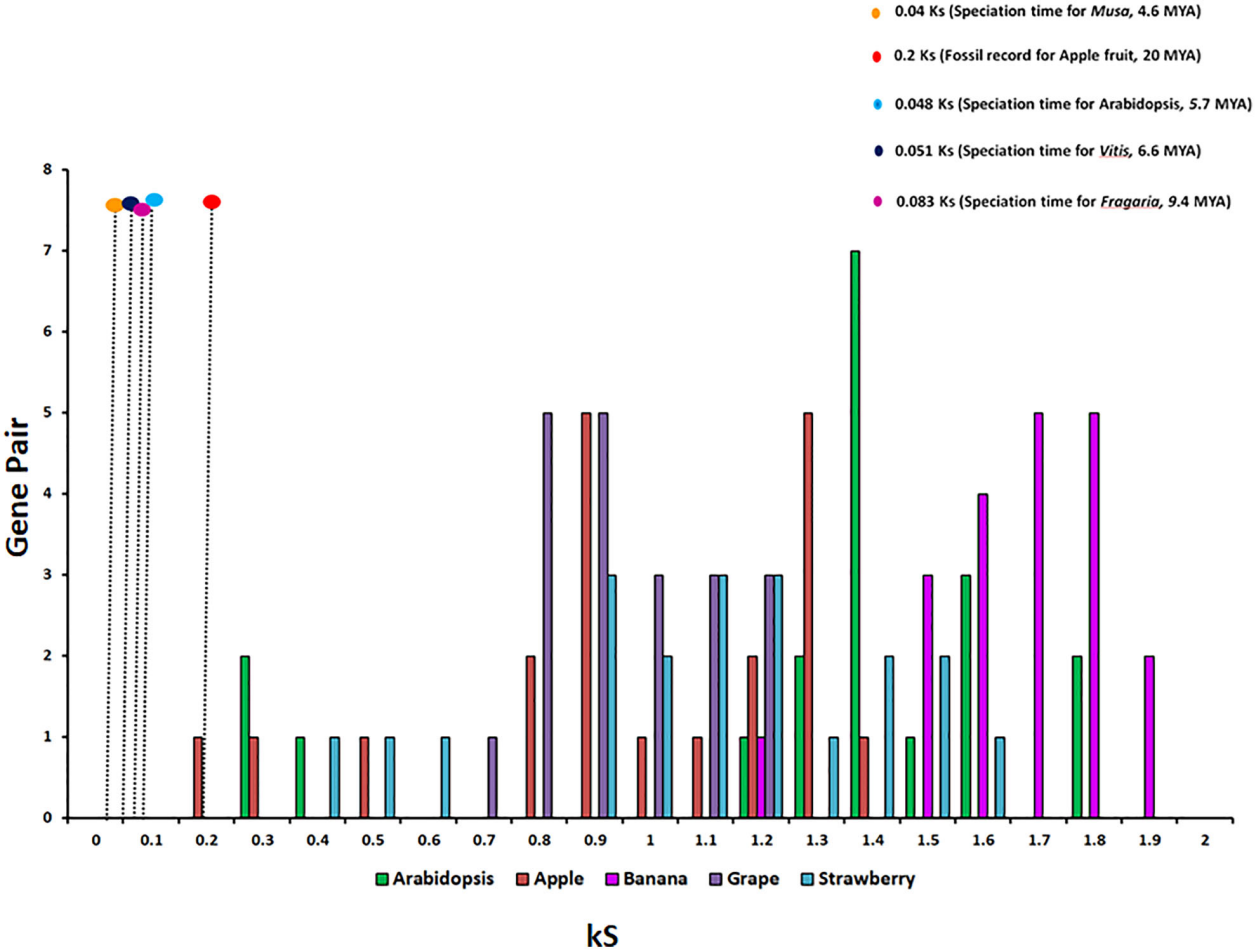
The relative substitution plot of kS score of gene pairs of fruit carbamoyltransferase genes, representing the synonymous substitution sites with speciation time of different fruits and *Arabidopsis*. The speciation time has been taken from timetree.org.

**Figure 4:**
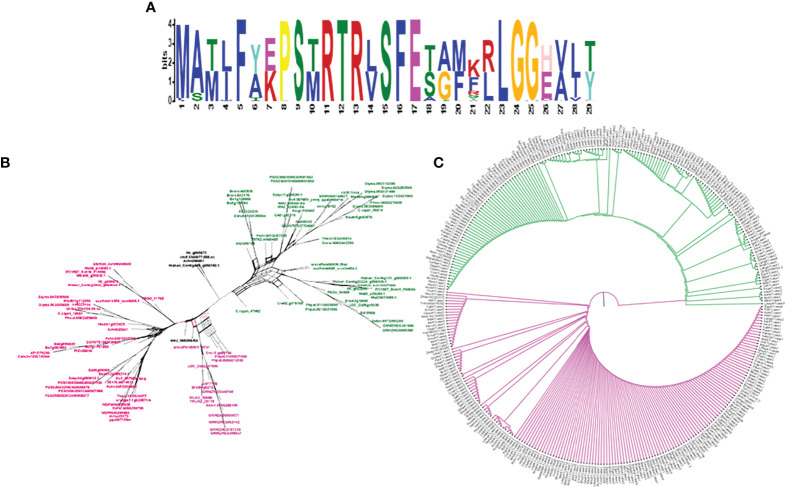
The logo representation of conserved motif **(A)** in carbamoyltransferase domain of plant groups and phylogeny **(C)** and network **(B)** of the same, representing the signature distribution of 2 different classes of carbamoyltransferase: aspartate carbamoyltransferase (Pink) and ornithine carbamoyltransferase (Green).

To study the phylogenetic relationship and evolutionary gains, the total identified 393 sequences and 98 representative sequences were aligned and subjected for maximum likelihood-based phylogeny using the IQ-TREE tool with best-fit model JTT + R6, chosen according to the Bayesian information criterion. The kingdom-wide phylogenetic tree (**Supplementary Figure S1**) showed a clear division between the ATCase and OTCase sequence signatures. This division defines the evolutionary conserved pattern of sequence signatures, where they retain the specific signature probably because of their functional diversity. To understand the division in signatures, the split phylogeny network analysis was performed using the SplitsTree4 tool. The obtained kingdom-wide phylogenetic network (**Supplementary Figure S2**) revealed the clear split between two different signatures and also showed the subsplit in both signatures rising from the basal signatures for both ATCase and OTCase. In both methods of phylogeny, tree construction and phylo-network, it was observed that there were different subbranches, following a mixed dicot–monocot pattern and a no climacteric versus non-climacteric pattern. To identify the pattern of similar signatures, the microsynteny analysis was performed using the SynNet pipeline. Interestingly, the microsynteny analysis (**Figure 1**) revealed the lineage-specific gene cluster formation in selected genes. This pattern of cluster formation indicates toward the shared lineage-specific signature. The lineage-specific pattern of shared sequence signatures reflects the plant clade–dependent evolutionary gain with maintaining the functional domain signature. A similar phylogenetic tree and split network (**Figure 2**) method was applied on 99 representative sequences, and similar representation was observed, where a distinct division was visible in ATCase and OTCase clades, supported and stabilized by mixed group subclades. This entire phylogeny and network analysis showed that both types of carbamoyltransferase are evolutionary-conserved and have lineage-specific sequence signatures, which have conserved amino acids to define sequence identity and variable amino acids for possible evolutionary and functional adaptation. The phylogeny of representative members reconfirmed that the carbamoyltransferase group maintained the functional identity in terms of domain composition and organization, with conserved amino acid sequence and gaining other features with variable amino acids. In microsynteny analysis, it was expected to get dicot- and monocot-specific clusters, but the clusters obtained were of mixed group and divided in shared sequence blocks according to aspartate and ornithine class. This shows that functional conservation of carbamoyltransferases are not affected by the dicot–monocot divergence. The fruit group members showed a similar observation, where no specific cluster was observed with higher number of fruit members or climacteric and non-climacteric gene clusters. To understand the evolutionary emergence of carbamoyltransferases in fruit groups, the sequence substitution analysis for synonymous (kS) and non-synonymous (kA) sites was performed (**Figure 3**). Initially, the substitution analysis was designed with *M. polymorpha* and *S. moellendorffii* as the evolutionary standard, but because of the highly fluctuating substitution divergence score, they were removed, and *Arabidopsis* was included as the evolutionary standard. In the fruit group, two climacteric fruits, apple and banana, and two non-climacteric fruits, grape and strawberry, were considered as fruit representative. The kS plot (**Figure 3**) was plotted with kS score against the gene pairs with speciation time of each fruit; the speciation time was obtained from timetree.org. The kS plot revealed that a significant amount of gene pairs of fruit groups emerges after the speciation time of selected fruits. The kS analysis also showed that the kS value is converged, which reflects the conserved pattern of substitution site selection. As all the analytical methods were indicating toward the conserved sequence signature, the conserved motif search was performed using MEME tool. The motif search result showed a 29-aa long motif (**Figure 4**), with conserve (M, F, P, S, R, T, R, S, F, E) and variable aa. The reconfirmation phylogeny was constructed using the motif sequence, and again, the similar result was obtained in both (phylogeny and split network). This clearly shows the highly conserve nature of carbamoyltransferase genes where specific aa are involved in maintaining the functional identity, whereas other aa are replaced in accordance with evolutionary adaptation. The motif phylogeny also showed the clear plant group–based subclade formation. Thus, the ATCase and OTCase genes have evolved throughout the plant kingdom with minimal sequence divergence.

To understand the functional impact of conserved sequential evolution on the structures of ATcase/OTcase proteins, the bacterial protein model of ATcase (*Escherichia coli*, PDB ID: 5vmq) was compared with five plant representative protein models of algae (*G. pectorale*), bryophyte (*M. polymorpha*), pteridophyte (*S. moellendorffii*), gymnosperm (*T. plicata*), and angiosperm (*A. thaliana*). The sequential comparison (**Supplementary Figure S3**) between the above-mentioned models showed significant variation in amino acid sequences with clear differences in bacterial conserved amino acids and plant conserved amino acids. The structural comparison (**Supplementary Figure S4**) shows highly conserved protein structure throughout the plant representatives. The comparison between bacterial and plant protein showed a significant structural enhancement, where the small helical structure of bacterial protein was replaced by the loop structure in plant protein models for functional adaptation. All the plant ATcases studied were highly conserved in sequence and structure.

### Transcriptomic expression and methylation status of aspartate carbamoyltransferase/ornithine carbamoyltransferase genes during fruit development and ripening

**Figure 5:**
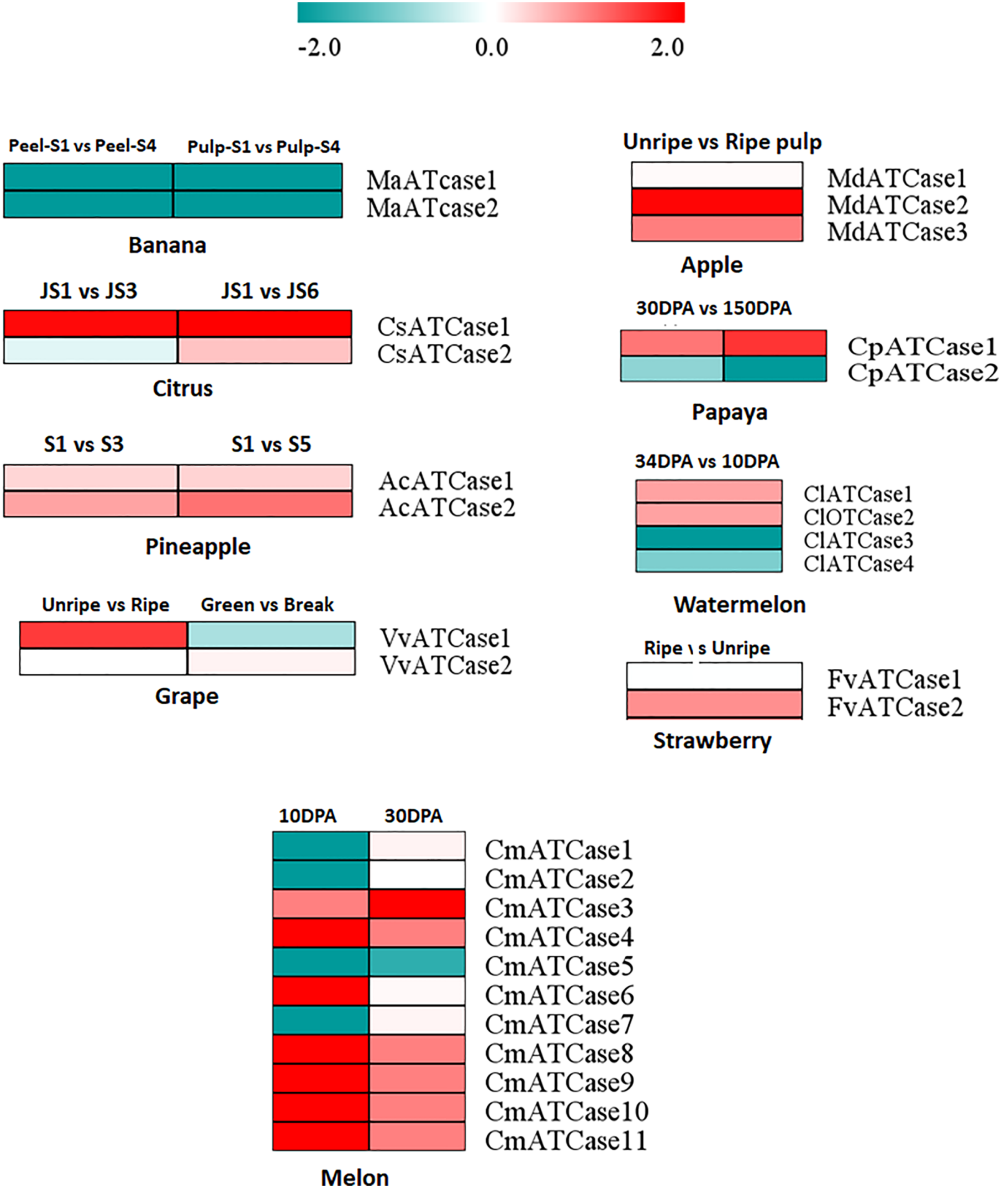
The heat map representation of the differential expression profile at the log2 fold change scale of carbamoyltransferase genes in different climacteric and non-climacteric fruits in different developmental and ripening conditions.

To study the expression of ATcase/OTcase genes, the geo profiles of transcriptomic data from fruit encode initiative were downloaded for different fruit developmental stages. The DE was considered from the log2 of fpkm values using the DEseq package and plotted as heat map. The heat map (**Figure 5**) showed a contrasting pattern of expression where one half of genes were showing a upregulation while the other half were showing downregulation. In most of the cases, the ATCase genes were upregulated, whereas the OTCase genes were downregulated. In case of banana, both genes showed downregulation. The expression analysis indicated the positive involvement of ATCase genes in fruit development and ripening without climacteric and non-climacteric specific expression grouping (Joshi et al., 2019).

**Figure 6:**
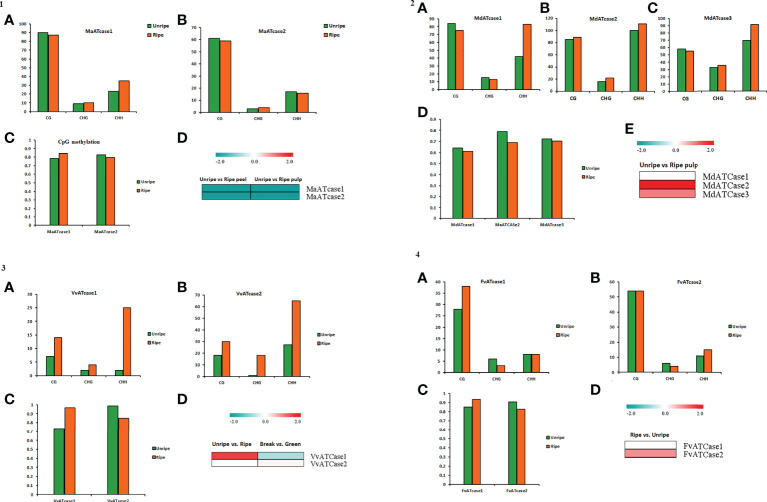
The panel representation for the status of CG, CHG, and CHH methylation sites in ATcase genes of banana (panel 1), apple (panel 2), grape (panel 3), and strawberry (panel 4). Status of CG, CHG, and CHH methylation sites in ATcase genes of banana, represented by bar plots **(A, B)** in unripe and ripe pulp conditions. Level of CpG methylation in banana Atcase genes **(C)**, represented by a bar plot, and compared with log2 fold change expression, represented by a heat map **(D)**. Status of CG, CHG, and CHH methylation sites in ATcase genes of apple, represented by bar plots **(A–C)** in unripe and ripe pulp conditions. Level of CpG methylation in banana Atcase genes **(D)**, represented by a bar plot, and compared with log2 fold change expression, represented by a heat map **(E)**. Status of CG, CHG, and CHH methylation sites in ATcase genes of grape, represented by bar plots **(A, B)** in unripe and ripe pulp conditions. Level of CpG methylation in grape Atcase genes **(C)**, represented by a bar plot, and compared with log2 fold change expression, represented by a heat map **(D)**. Status of CG, CHG, and CHH methylation sites in ATcase genes of strawberry, represented by bar plots **(A, B)** in unripe and ripe pulp conditions. Level of CpG methylation in strawberry Atcase genes **(C)**, represented by a bar plot, and compared with log2-fold change expression, represented by a heat map **(D)**.

To explore the role of cytosine methylation and related changes, the bs-seq data, provided by a fruitENCODE initiative, was used and mapped against the genomic coordinates. The obtained mapping result was further processed for the methylation status and methylation context for the ATcase/OTcase genes. The identified methylation context (CpG, CHG, and CHH) with significant total methylation levels was calculated for the unripe and ripe conditions using a CLC genomics workbench with default parameters. The methylation contexts and values were represented by a bar plot, along with the transcriptomic expression, to understand the correlation between them, if any (**Figure 6**). In methylation analysis, it was observed that, in case of climacteric representatives, banana (**Figure 6**) and apple (**Figure 6**), the CG methylation level was lower in the ripe condition, whereas in case of non-climacteric representatives, grape (**Figure 6**) and strawberry (**Figure 6**), the CG methylation level was higher during the ripe condition. Another interesting observation revealed that, in case of apple and grape, the CHH methylation level is much higher in the ripe condition in comparison with the unripe condition, with banana and strawberry. Though there are previous reports in tomato and citrus (Huang et al., 2019; Li et al., 2022; Liu et al., 2022), which showed the active involvement and global effect of methylation during fruit developmental stages and ripening, our analysis partially matched with them in case of banana apple and grape. In case of banana (**Figure 6**), the CG methylation level was equally higher for both genes in the unripe and ripe conditions in pulp and their transcriptomic expression was down, whereas in case of apple (**Figure 6**), again the overall CG methylation level was high and with a slight difference between the unripe and ripe conditions; the transcriptomic expression was upregulated. As mentioned earlier, that in case of apple and grape the CHH methylation level was higher, it is possible that the CHH methylation level is balancing or accompanying the CH levels and actively regulating the fruit ripening, which was earlier observed in case of pepper fruit (Xiao et al., 2020), citrus (Huang et al., 2019), and tomato (Liu et al., 2022). The methylation status of grape (**Figure 6**) showed a higher CG and CHH context, similar to apple, while the CG methylation level was higher in the VvATcase1 gene during the ripe condition, and the same gene was upregulated during the ripe condition; the other gene, VvATcase2, showed a lower CG level in the ripe condition and a very small increase in expression during the ripe condition. In case of strawberry, the methylation status of the CG context was much higher in comparison with the rest of the two contexts. The CG methylation level of the FvATcase1 gene (**Figure 6**) was higher in the ripe condition and transcriptomic expression was very low, whereas in case of FvATcase2, the negatively correlated pattern was observed where CG level was lower in the ripe condition and trancriptomic expression was positively higher. Overall, the methylation status of ATcase/OTcase genes during fruit development and ripening was partially following the patterns, in accordance with previous reports (Gallusci et al., 2016; Tang et al., 2020; Jia et al., 2022; Li et al., 2022).

### Co-expression network during ripening

**Figure 7:**
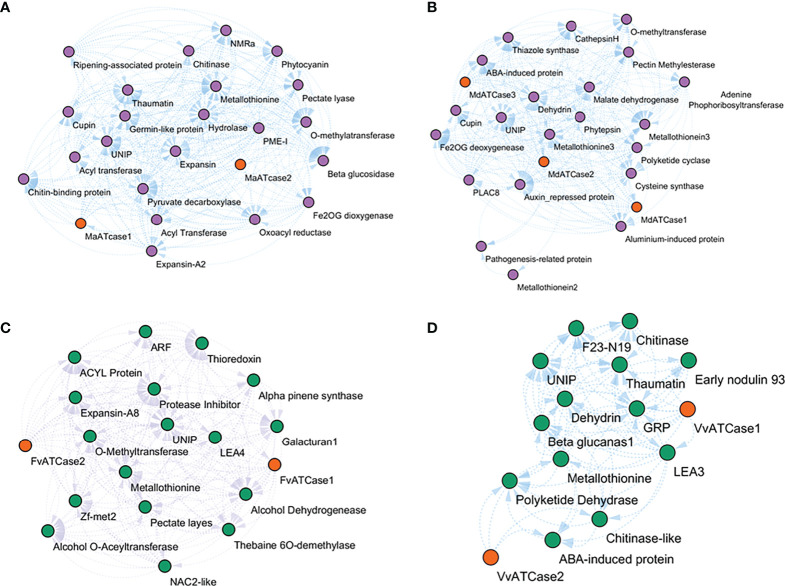
Relative co-expression network of aspartate and ornithine carbamoyltransferase genes during fruit ripening condition in banana **(A)**, apple **(B)**, grape **(C)**, and strawberry **(D)**.

The co-expressing genes during ripening were identified by calculating the expression correlation matrix. The result was plotted in a force-directed layout using the correlation value as fusion value for the significant nodes, and ATcase genes were represented by the contrasting color balls. The co-expression network was calculated for the climacteric (banana and apple) and non-climacteric (grape and strawberry) representatives (**Figure 7**). The co-expression network were assumed to reflect the difference of climacteric and non-climacteric machinery in fruit ripening, but the retrieved co-expression network showed a conserved gene network pattern.

The co-expression network reflected the conserved functional nature of the carbamoyltransferase group in fruit ripening. The genes that participate in different stages and steps of fruit ripening were observed in co-expression networks of banana, apple, grape, and strawberry. Genes like expansin (Jiang et al., 2019), pectate lyase (Uluisik and Seymour, 2020), metallothionine (Liu et al., 2002), methyltransferase, beta-glucosidase, hydrolase (Yao et al., 2014), and alcohol dehydrogenase (Speirs et al., 1998) were observed in correlation with ATcase/OTcase genes. This shows that the basic regulatory involvement of carbamoyltransferases has a potential role in fruit ripening. Also the ABA-induced protein LEA (Jeon et al., 2006) and dehydrin-like genes were observed, which might be an indication of the ABA-mediated regulation of fruit ripening and thus are co-expressed. No gene of polyamine pathway during ripening was observed in the co-expression network. Overall, the co-expression network revealed the conserved functional network of ATcase/OTcase genes during fruit development and ripening.

## Conclusion

The carbamoyltransferase genes were identified from the large number of plant genomes focusing on fruit ripening. Though the members showed variation in amino acid sites, the main functional sequence signature was highly conserved for both types of representatives, along with their phylogenetic signature splitting and conserved motif. The microsynteny analysis showed the lineage-specific conservation of these genes. The expression of these genes showed, especially the aspartate carbamoyltransferase, positive regulation during fruit ripening, along with the influence of methylation and conserved co-expressing gene network. The carbamoyltransferase genes play a crucial role in various biological processes; thus, this study is important in understanding their evolutionary conserved and related functional features.

## Data availability statement

The datasets presented in this study can be found in online repositories. The names of the repository/repositories and accession number(s) can be found in the article/**Supplementary Material**.

## Author contributions

YD and MA conceptualized the study. YD collected the data and carried out the analysis. MA supervised the study. YD and MA wrote the manuscript. All authors contributed to the article and approved the submitted version.

## Funding

The Council of Scientific and Industrial Research, New Delhi, is acknowledged for their financial support (GAP 3441 and MLP006).

## Acknowledgments

The authors also acknowledge the CSIR-4PI Institute for providing the supercomputing facility. Manuscript number: CSIR-NBRI_MS/2022/09/01.

## Conflict of interest

The authors declare that the research was conducted in the absence of any commercial or financial relationships that could be construed as a potential conflict of interest.

## Publisher’s note

All claims expressed in this article are solely those of the authors and do not necessarily represent those of their affiliated organizations, or those of the publisher, the editors and the reviewers. Any product that may be evaluated in this article, or claim that may be made by its manufacturer, is not guaranteed or endorsed by the publisher.

## Supplementary material

Supplementary Figure 1 Kingdom-wide phylogenetic tree of carbamoyltransferase genes, showing the major clades in plants groups for aspartic carbamoyltransferase (Pink) and ornithine carbamoyltransferase genes (Green). The basal groups are in represented by blue colour.

Supplementary Figure 2 Kingdom-wide phylogenetic network splitting of carbamoyltransferase genes, showing the major sequential splits in plants groups for aspartate carbamoyltransferase (Red and pink) and ornithine carbamoyltransferase genes (Green and blue). The basal plant sequence signatures are located in center.

Supplementary Figure 3 Multiple sequence alignment between the bacterial, algal, bryophyte, pteridophyte, gymnosperm and angiospermic protein model representatives, for conserve amino acid pattern.

Supplementary Figure 4 The tile representation to compare the protein structures of the bacterial, algal, bryophyte, pteridophyte, gymnosperm and angiospermic protein model representatives.
