# Supplementary figures and images for: Genome and transcriptome-wide study of carbamoyltransferase genes in major fleshy fruits: A multi-omics study of evolution and functional significance
Source: Front Plant Sci. 2022 Nov 3;13:994159. doi: 10.3389/fpls.2022.994159 (PMC9669488; doi:10.3389/fpls.2022.994159)

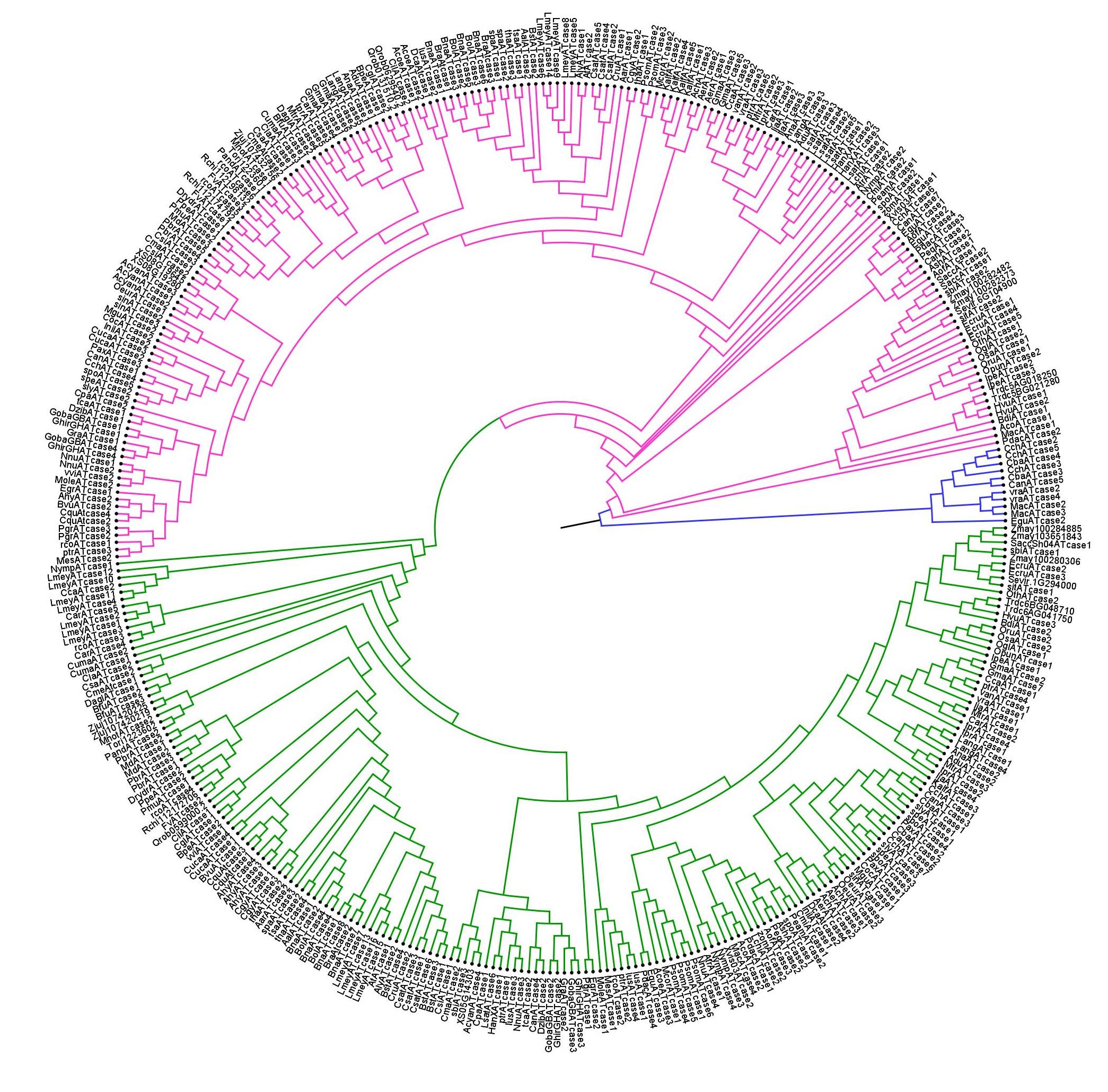

Supplement: Supplementary Figure 1 — Kingdom-wide phylogenetic tree of carbamoyltransferase genes, showing the major clades in plants groups for aspartic carbamoyltransferase (Pink) and ornithine carbamoyltransferase genes (Green). The basal groups are in represented by blue colour. [file Image_1.jpeg]

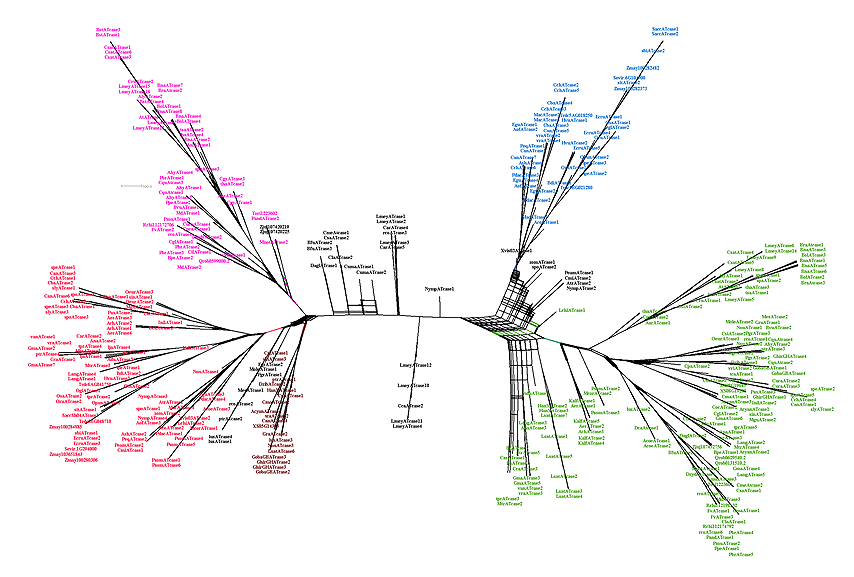

Supplement: Supplementary Figure 2 — Kingdom-wide phylogenetic network splitting of carbamoyltransferase genes, showing the major sequential splits in plants groups for aspartate carbamoyltransferase (Red and pink) and ornithine carbamoyltransferase genes (Green and blue). The basal plant sequence signatures are located in center. [file Image_2.png]

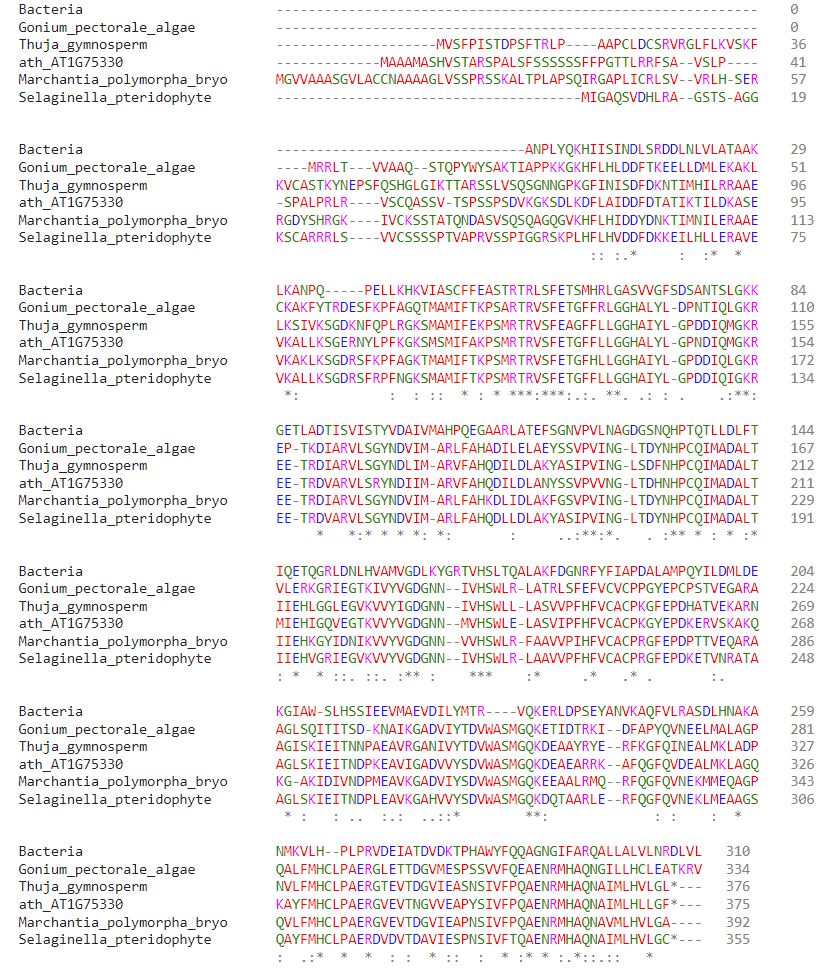

Supplement: Supplementary Figure 3 — Multiple sequence alignment between the bacterial, algal, bryophyte, pteridophyte, gymnosperm and angiospermic protein model representatives, for conserve amino acid pattern. [file Image_3.jpeg]

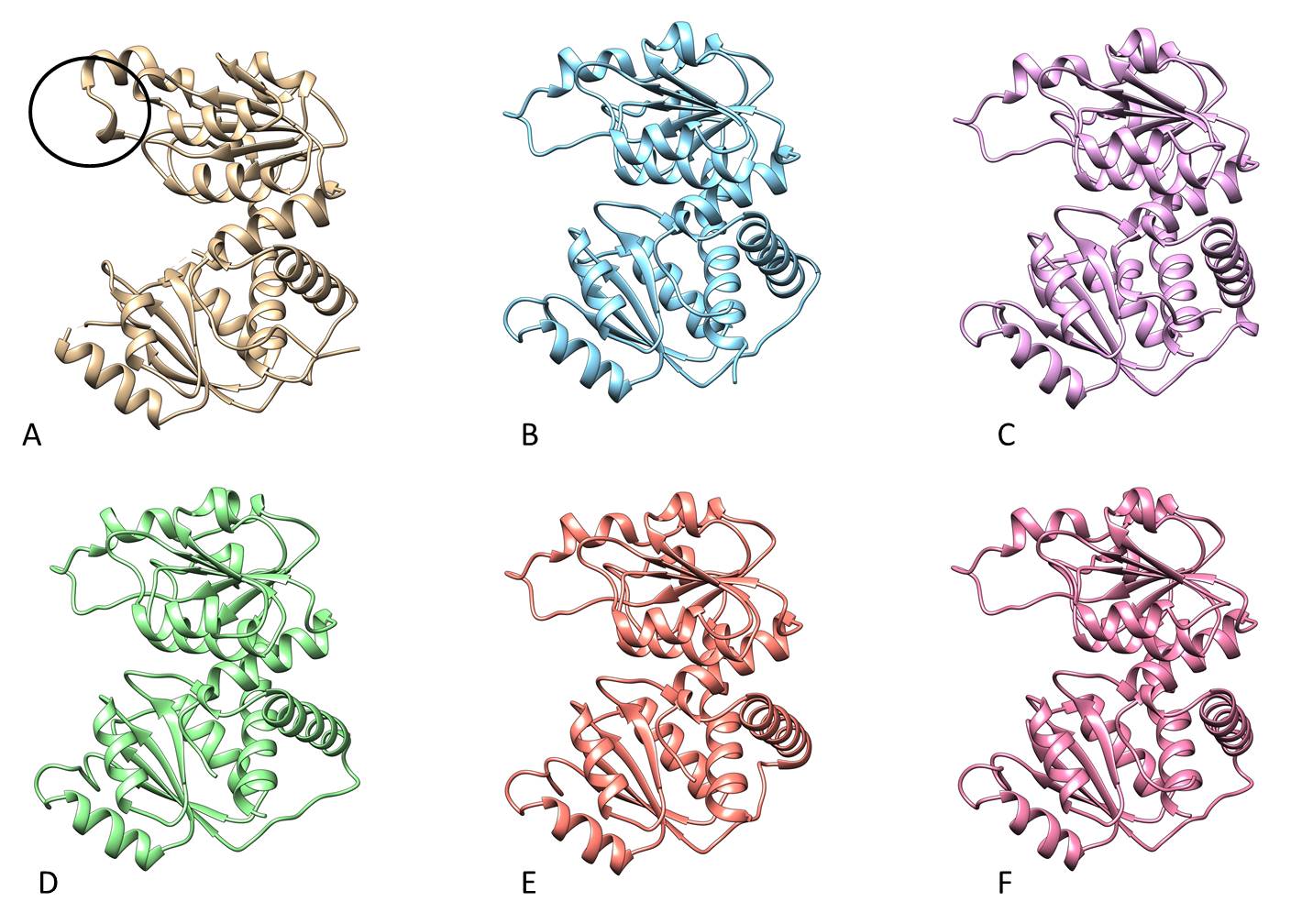

Supplement: Supplementary Figure 4 — The tile representation to compare the protein structures of the bacterial, algal, bryophyte, pteridophyte, gymnosperm and angiospermic protein model representatives. [file Image_4.jpeg]
